# Recycled Aggregates for Sustainable Construction: Strengthening Strategies and Emerging Frontiers

**DOI:** 10.3390/ma18133013

**Published:** 2025-06-25

**Authors:** Ying Peng, Shenruowen Cai, Yutao Huang, Xue-Fei Chen

**Affiliations:** 1 School of Civil Engineering, Putian University, Putian 351100, China; 2 Engineering Research Center of Disaster Prevention and Mitigation of Southeast Coastal Engineering Structures (JDGC03), Fujian Province University, Putian 351100, China

**Keywords:** construction wastes, recycled aggregates, concrete, waste valorization, sustainable urban development

## Abstract

The transformative trajectory of urban development in the contemporary era has engendered a substantial escalation in construction waste generation, particularly in China, where it constitutes approximately 40% of the total solid waste stream. Traditional landfill disposal methodologies pose formidable ecological challenges, encompassing soil contamination, groundwater pollution, and significant greenhouse gas emissions. Furthermore, the unsustainable exploitation of natural sandstone resources undermines energy security and disrupts ecological balance. In response to these pressing issues, an array of scholars and researchers have embarked on an exploratory endeavor to devise innovative strategies for the valorization of construction waste. Among these strategies, the conversion of waste into recycled aggregates has emerged as a particularly promising pathway. However, the practical deployment of recycled aggregates within the construction industry is impeded by their inherent physico-mechanical properties, such as heightened water absorption capacity and diminished compressive strength. To surmount these obstacles, a multitude of enhancement techniques, spanning physical, chemical, and thermal treatments, have been devised and refined. This paper undertakes a comprehensive examination of the historical evolution, recycling methodologies, and enhancement strategies pertinent to recycled aggregates. It critically evaluates the efficacy, cost–benefit analyses, and environmental ramifications of these techniques, while elucidating the microstructural and physicochemical disparities between recycled and natural aggregates. Furthermore, it identifies pivotal research gaps and prospective avenues for future inquiry, underscoring the imperative for collaborative endeavors aimed at developing cost-effective and environmentally benign enhancement techniques that adhere to the stringent standards of contemporary construction practices, thereby addressing the intertwined challenges of waste management and resource scarcity.

## 1. Introduction

Since the advent of the new era, the landscape of urban development has undergone a transformative evolution, characterized by the swift progression of economic construction endeavors and the relentless push towards urbanization [1]. This period has witnessed the emergence of novel architectural designs, which, in turn, has given rise to a deluge of construction waste, posing significant challenges to sustainable urban management. Inorganic construction materials contribute to soil and groundwater contamination through mechanisms such as heavy metal leaching from concrete and mortar, alkaline runoff from cement-based products, particulate deposition of construction dust, and leachate generation in improperly managed landfills; these processes introduce pollutants like lead, chromium, sulfates, and chlorides into ecosystems, degrading soil fertility, poisoning groundwater aquifers, and posing long-term risks to human health and biodiversity, necessitating mitigation through sustainable material use, strict waste management, and advanced remediation technologies.

In the context of China, the magnitude of construction waste is particularly alarming, accounting for approximately 40% of the total solid waste generated nationwide [2]. This statistic underscores the pressing need for innovative strategies to address the burgeoning waste problem. Traditionally, the predominant approach to managing construction waste has been landfill disposal. While this method may seem expedient, it imposes considerable ecological strain and contributes to the wastage of potentially valuable resources. The ecological footprint of landfill sites, including soil contamination, groundwater pollution, and greenhouse gas emissions, further exacerbates the issue. Concurrently, the exponential growth of new buildings has led to a relentless surge in concrete consumption, resulting in the overexploitation of natural sandstone resources. This unsustainable trend not only threatens energy security but also precipitates secondary ecological damage, such as habitat destruction and biodiversity loss.

In response to these multifaceted challenges, scholars and researchers have delved into exploring alternative pathways for the utilization of construction waste. One promising avenue is the conversion of construction waste into recycled aggregates through advanced processing techniques [3,4,5]. This transformation not only mitigates the environmental burden associated with landfill disposal but also harnesses the waste as a secondary resource, thereby fostering circular economy principles. However, the practical application of recycled aggregates in the construction industry is not without its hurdles. One of the principal limitations is their inherent physical properties, particularly their high water absorption rate and relatively low strength, which can compromise the structural integrity and durability of concrete produced using these aggregates. These attributes necessitate the development of effective enhancement strategies to elevate the performance of recycled aggregates to levels comparable or superior to those of natural aggregates.

The enhancement of recycled aggregates is a multifaceted endeavor that involves a variety of techniques aimed at improving their mechanical properties [6], durability [7], and compatibility with cementitious materials. These techniques can be broadly classified into physical, chemical, and thermal treatments. Physical treatments, such as mechanical crushing and screening, are employed to refine the particle size distribution and remove contaminants. Chemical treatments, including the application of chemical admixtures and surface coatings, aim to modify the surface chemistry of the aggregates, reducing water absorption and enhancing bond strength with cementitious matrices. Thermal treatments, on the other hand, involve exposing the aggregates to high temperatures to alter their mineral composition and microstructure, thereby improving their mechanical properties. Despite the advancements made in recycled aggregate enhancement technologies, several challenges persist. The optimization of treatment parameters to achieve desired aggregate properties without compromising cost-effectiveness remains a significant hurdle [8,9]. Additionally, the long-term performance and environmental impact of enhanced recycled aggregates in real-world applications require further investigation. Moreover, the integration of these aggregates into existing construction practices necessitates the development of standardized testing protocols and regulatory frameworks to ensure safety and compliance.

To address these challenges and advance the field, this paper undertakes a comprehensive review of the historical evolution and recycling processes of recycled aggregates. It delves into the microstructural and physicochemical differences between recycled and natural aggregates, highlighting the underlying mechanisms that govern their performance in concrete. Building upon this foundation, this paper provides an exhaustive overview of the current enhancement methodologies for recycled aggregates, evaluating their effectiveness, cost–benefit ratios, and potential environmental implications. Furthermore, it identifies the key research gaps and future directions in this domain, aiming to stimulate further innovations and collaborations among stakeholders. Overall, the transformation of construction waste into recycled aggregates presents a viable solution to the pressing waste management and resource scarcity challenges facing the construction industry. However, the full realization of this potential necessitates concerted efforts in developing cost-effective, eco-friendly enhancement techniques that can elevate the performance of recycled aggregates to meet the standards of modern construction practices. Through research, innovative technological advancements, and collaborative stakeholder engagement, the vision of a circular economy in the construction sector can be brought closer to reality.

## 2. Recycled Aggregates: Generation, Recycling, and Characterization

### 2.1. The Generation of Recycled Aggregates

During the Second World War, the extensive devastation of infrastructure, including buildings and roadways, necessitated an urgent post-war reconstruction effort coupled with waste removal. This unique juncture in history led to the nascent utilization of demolished construction debris as recycled aggregates, primarily in Germany and the United Kingdom. The emergence of this practice marked a pivotal shift in resource management and waste reduction strategies. However, the trajectory of recycled aggregate adoption was not linear. Post-war, its use underwent a gradual decline until the 1970s, when the United States spearheaded a resurgence by reintroducing recycled aggregates in non-structural concrete applications, such as foundational supports, base layers, and fill materials. Concurrently, the United Kingdom witnessed a progressive annual increase in the recovery and utilization of recycled aggregates, culminating in the pioneering use of recycled coarse aggregates in the production of novel structural concretes for construction purposes [10,11,12]. This transformation signified the ascendancy of recycled aggregates as a subject of scientific scrutiny and sustainable development discourse.

In parallel, Germany and Japan emerged as forefront nations in the realm of construction waste utilization, achieving utilization rates surpassing 50%. These countries have accumulated substantial expertise in construction waste management technologies. Germany, in particular, holds a historic distinction as the first nation to embark on the recycling and resource recovery of construction waste. With governmental support, Germany achieved a commendable recycling rate of 18% for waste concrete in 1994 and subsequently issued the “Guidelines for the Application of Recycled Concrete Aggregates” in August 1998 [13]. This comprehensive document stipulates stringent specifications for the composition, grading, mineralogy, water absorption capacity, crushing value, and other pertinent performance indicators of recycled aggregates, as well as the resultant concrete properties. This groundbreaking publication catalyzed steady advancements in the development and application of recycled aggregate concretes in Germany, culminating in a series of notable achievements.

Japan, faced with resource scarcity, has demonstrated a heightened commitment to the recycling of construction waste. Early on, it formulated a robust suite of technical specifications and standards tailored to construction waste. In 1994, Japan unveiled usage guidelines for recycled aggregates and recycled concrete, delineating their applications and utilization requirements [14]. These guidelines were instrumental in elevating the utilization rate of construction waste, thereby fostering sustainable resource management. With the successive promulgation of policies, Japan swiftly ascended to the forefront of construction waste recycling. Currently, Japan boasts a sophisticated research framework examining the water absorption, mechanical strength, durability, and other material properties of recycled aggregates. This rigorous investigative endeavor has laid a solid foundation for the efficient and sustainable utilization of construction waste.

In conclusion, the substitution of natural aggregates with recycled aggregates in novel building structures embodies a paradigm shift towards environmental stewardship and human sustainable development. This evolution underscores the significance of leveraging scientific advancements and policy frameworks to enhance the circular economy and mitigate the adverse impacts of construction waste on the natural environment.

### 2.2. In-Depth Analysis of the Recycling Process for Recycled Aggregates

The generation of construction waste is a significant concern, primarily occurring during the phases of construction, renovation, and demolition. Among these, demolition generates the highest total volume of construction waste [15,16]. In the context of recycling recycled aggregates, the initial step involves pre-crushing. However, subsequent to mechanical crushing, these aggregates exhibit a pronounced degree of heterogeneity, posing challenges in their utilization. To address this heterogeneity issue, Ambrós et al. [17,18] introduced a sophisticated density classification method. This method leverages the wall effect within an air-jitter device to effectively separate particles. The air-jitter device comprises several integral components: a feed inlet, a separation chamber, an operation center, and dust filtration systems. During the separation process, the material is propelled by dual airflows, causing it to collide with the wall surface and subsequently fall into distinct rectangular cross-sections. This method facilitates the separation of particles, ultimately enhancing the quality of the recycled aggregates.

Xu et al. [19] further contributed to this field by adopting an innovative process involving the dispersion and filling of recycled aggregate concrete. This approach aims to improve the uniformity of the aggregates, thereby reducing the size of the old interfacial transition zone and enhancing their compactness. By doing so, the overall performance of the recycled aggregates is significantly improved. Khoury et al. [20] conducted a comprehensive analysis of the homogenization process using a loader. They employed multiple loading/unloading cycles to spread the aggregates into a nearly circular platform and subsequently divided it into zones using a grid system. Within each zone, a planetary mixer was utilized to crush the aggregates. Finally, homogenization was achieved in a closed container, resulting in improvements in the water absorption and compactness of the recycled aggregates. These enhancements led to a notable improvement in the physical properties and durability of the recycled aggregate concrete.

To further refine the recycling process, it is imperative to employ methods that not only homogenize the crushed materials but also address the heterogeneity issue at its source. This can be achieved by incorporating advanced techniques for removing or strengthening the hardened paste on the aggregate surface. By doing so, the performance of the recycled concrete can be maximized, ensuring its suitability for various applications. In conclusion, the recycling process for recycled aggregates involves multiple stages, each requiring careful consideration and precision. By leveraging advanced techniques such as density classification, dispersion and filling processes, and homogenization using loaders and planetary mixers, it is possible to obtain high-quality recycled aggregates. However, to fully harness the potential of recycled aggregates, it is crucial to combine these methods with innovative approaches for addressing the heterogeneity issue at its source. This holistic approach will ultimately lead to the development of recycled concrete with superior performance characteristics.

### 2.3. Distinct Characteristics Between Recycled Aggregates and Natural Aggregates

**Figure 1:**
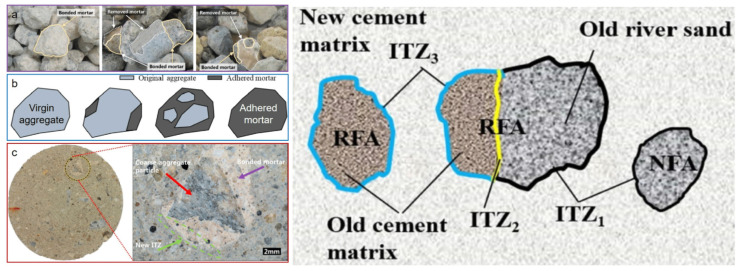
Interfacial transition zones (ITZs) of the recycled aggregates: multi-scale morphology (**a**–**c**) [23] and multiple ITZs [24]. Note: RFA—recycled fine aggregate; NFA—natural fine aggregate.

Recycled aggregates, which primarily encompass hardened cement mortar, virgin aggregates, and the intricate interfacial transition zones (ITZs) that exist between these constituents [21,22], exhibit a unique structural composition, as delineated in Figure 1. This figure illustrates the varied structural configurations of different types of recycled aggregates. The primary differentiation between recycled and natural aggregates stems from the inclusion of two additional, intrinsic components within recycled aggregates: the hardened cement paste adhering to their surfaces and the old ITZs between this paste and the aggregates. These components play pivotal roles in determining the overall performance of recycled aggregates.

Recycled aggregates, after crushing, often manifest heterogeneities, large porosity, dense microcracks, and low crushing values. Consequently, they exhibit elevated water absorption rates, reduced densities, and inferior mechanical strengths when compared to their natural counterparts. This phenomenon can be attributed to the intrinsic defects inherent in recycled aggregates. Wang et al. [25] conducted an in-depth investigation into the ramifications of failure modes and internal characteristics of recycled aggregates on concrete performance and examined the alterations in the mechanical attributes of the ITZs between aggregates and hardened cement paste. Their findings revealed marked disparities in microhardness between the old and new ITZs. Furthermore, the adherence of mortar to the aggregate surfaces was identified as a significant factor contributing to a substantial decrement in the performance of recycled aggregates.

From an application perspective, the inherent flaws within recycled aggregates can give rise to inaccuracies in the incorporation of mixing water, thereby exacerbating issues such as impaired workability in fresh concrete. In comparison to natural aggregate concrete, the old ITZs within recycled aggregates are notably weaker than their new counterparts. This predisposition makes recycled aggregates more susceptible to detachment from the surrounding new mortar. Such detachment leads to a decrease in the density of recycled concrete; an augmentation in water absorption rates; and ultimately, a decline in the mechanical properties of the resultant concrete. Consequently, as the proportion of recycled aggregates increases within a concrete mix, there is a corresponding decrement in the physical properties, mechanical attributes, and durability of the recycled concrete.

To address the aforementioned challenges associated with recycled aggregates, scholars globally have embarked on extensive research endeavors aimed at repairing and treating the mortar adhering to the surfaces of recycled aggregates and old ITZs. The ultimate objective of these endeavors is to bring the performance characteristics of recycled aggregates in closer alignment with those of natural aggregates. These research endeavors have employed advanced methodologies and analytical frameworks to enhance the understanding of the complexities involved and to develop innovative solutions that can mitigate the adverse effects associated with the use of recycled aggregates in concrete production.

## 3. Modification of Recycled Aggregates

In an in-depth review and analytical comparison of the performance disparities between recycled aggregates and their natural counterparts, it has emerged that the adhering mortar and the old interfacial transition zones (ITZs) exert a predominant influence on the overall properties of recycled aggregates. These components, inherent to recycled aggregates, introduce complexities that necessitate targeted interventions to enhance their utility in construction applications. In recognition of this, scholars have embarked on extensive research endeavors to devise methodologies aimed at mitigating the adverse effects associated with adhering mortar and ITZs. One such approach involves the removal of the mortar adhering to the surface of the aggregates. This technique seeks to eliminate the source of increased porosity and reduced mechanical strength that is often observed in recycled aggregates. Alternatively, researchers have explored methods for reinforcing the adhering mortar to augment its structural integrity and thereby improve the overall performance of the recycled aggregates.

### 3.1. Removal of Adhering Mortar

Effective methods for removing adhering mortar include (1) acid treatment, (2) mechanical grinding, (3) thermal treatment, (4) thermal grinding, and (5) autogenous cleaning processes. Some scholars have also employed combinations of these methods in an attempt to achieve even better strengthening effects.

#### 3.1.1. Acid Treatment for the Modification of Recycled Aggregates

The hardened cement paste adhering to the surface of recycled aggregates constitutes a complex, rigid structure formed through intricate chemical reactions between cement and water. This presents a formidable challenge in terms of surface treatment, as the removal of this paste is crucial for improving the overall quality and performance of the aggregates in construction applications. Recent research has revealed that selected acidic solutions possess the capacity to dissolve the hardened cement paste on the surface of recycled aggregates, thereby enhancing their properties [26]. Various factors, including the concentration and type of acid solution, influence the dissolution efficiency of the hardened cement paste. Commonly employed acidic solutions encompass acetic acid (CH_3_COOH), hydrochloric acid (HCl), sulfuric acid (H_2_SO_4_), and phosphoric acid (H_3_PO_4_), each with unique characteristics and dissolution mechanisms.

In the context of acid treatment for recycled aggregates, Thaue et al. [27] conducted an innovative study in which the aggregates were initially soaked in acetic acid (CH_3_COOH) solution. This soaking process weakened the bond between the hardened cement paste and the aggregate surface, facilitating subsequent mechanical grinding and removal of the paste. Chauhan et al. [28] further extended this approach by combining acid treatment with thermal processing. Their results indicated that preheating the aggregates to 600 °C and then immersing them in 0.7 mol/L HCl solution effectively dissolved the hardened cement paste, leading to a significant enhancement in the aggregate’s performance. Notably, the chloride ion (Cl^−^) content decreased with increasing thermal load, mitigating the potential environmental threat posed by Cl^−^ and reducing the risk of water pollution.

Despite these promising outcomes, the application of acid treatment for recycled aggregates is not without challenges. Post-treatment measures are essential to address the chemical impurities’ residues on the aggregates and the contaminated water generated during the process. The washing of aggregates to remove impurities necessitates substantial water consumption, while the disposal of chemically contaminated water, primarily composed of sulfates and chlorides, poses significant environmental risks if not properly treated. In the current context of water scarcity, these limitations pose substantial constraints on the widespread adoption of this enhancement method. Therefore, ongoing research is focused on developing more efficient and environmentally friendly treatments for recycled aggregates, aiming to balance performance enhancement with sustainability.

#### 3.1.2. Mechanical Grinding Treatment

**Figure 2:**
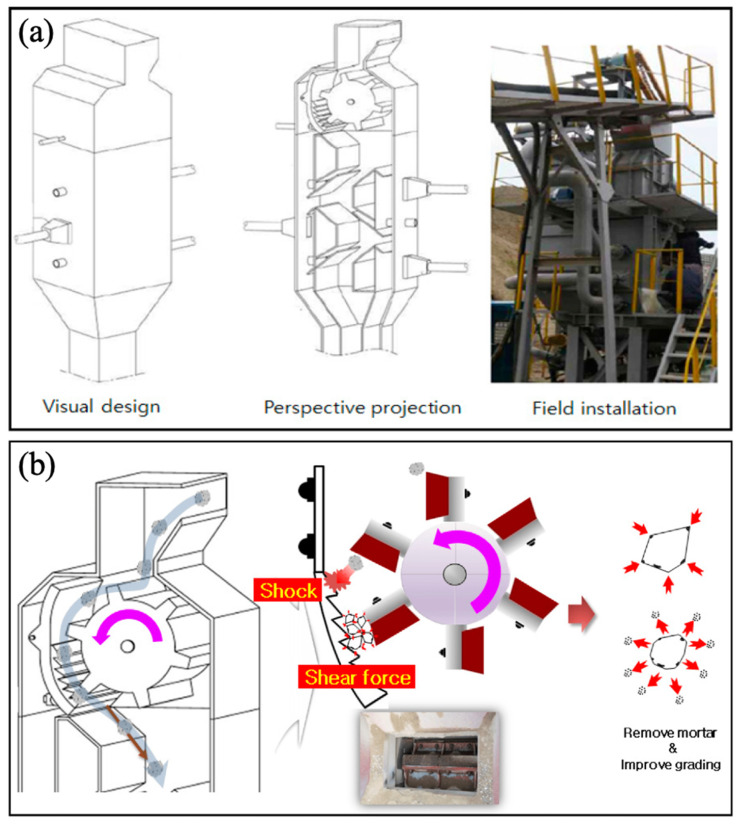
Mechanical removal of old mortar: (**a**) schematic diagram and the on-site image of the removal equipment; (**b**) mortar removal process [29].

Mechanical grinding treatment is a conventional method employed to eliminate hardened cement paste from the surface of recycled aggregates, leveraging the rolling action imparted by high-speed rotating eccentric gears within a grinding apparatus. As depicted in Figure 2, this procedure typically commences with immersing the recycled aggregates in water to soften the adhering mortar. Subsequently, the aggregates undergo a collision process against the wall, facilitated by the high-speed rotating eccentric gears, which induces shear stress conducive to the detachment of the surface paste [29]. With the evolution of traditional methodologies, corresponding advancements have been witnessed in grinding technologies. Notably, previous studies [30,31] introduced the utilization of a ball mill as an alternative to conventional machinery for treating the cement paste adhered to recycled aggregates. Subsequent performance evaluations affirmed the viability of this innovative approach. In comparison to conventional grinding, the ball mill exhibited superior efficiency and efficacy in eradicating the adhered mortar. Encouraged by these findings, researchers embarked on exploring hybrid methodologies that amalgamate ball milling with grinding, to identify even more effective treatment strategies.

However, the mechanical grinding process is not devoid of challenges. Firstly, the crushing action targeting the removal of adhered cement paste frequently results in the formation of numerous microcracks and pores on the aggregate surface. This phenomenon enhances the water absorption capacity of the recycled aggregates, ultimately impacting their durability and mechanical characteristics. Secondly, the high-speed operation of the grinding apparatus generates considerable heat while consuming substantial energy, leading to low energy utilization efficiency and elevated energy consumption. Hence, there exists a pressing need for further refinements in mechanical grinding treatment methodologies.

#### 3.1.3. Thermal Treatment

Traditional thermal treatment strengthening involves heating recycled aggregates to temperatures of approximately 500 °C for approximately two hours. This process exploits the thermal stress generated by the thermal expansion effect to fracture the surface paste, thereby facilitating the removal of the adhered mortar [32]. While this method imposes stress on the surface-hardened paste, it concurrently introduces stress to the aggregates themselves, causing the generation and propagation of microcracks on the aggregate surface.

**Figure 3:**
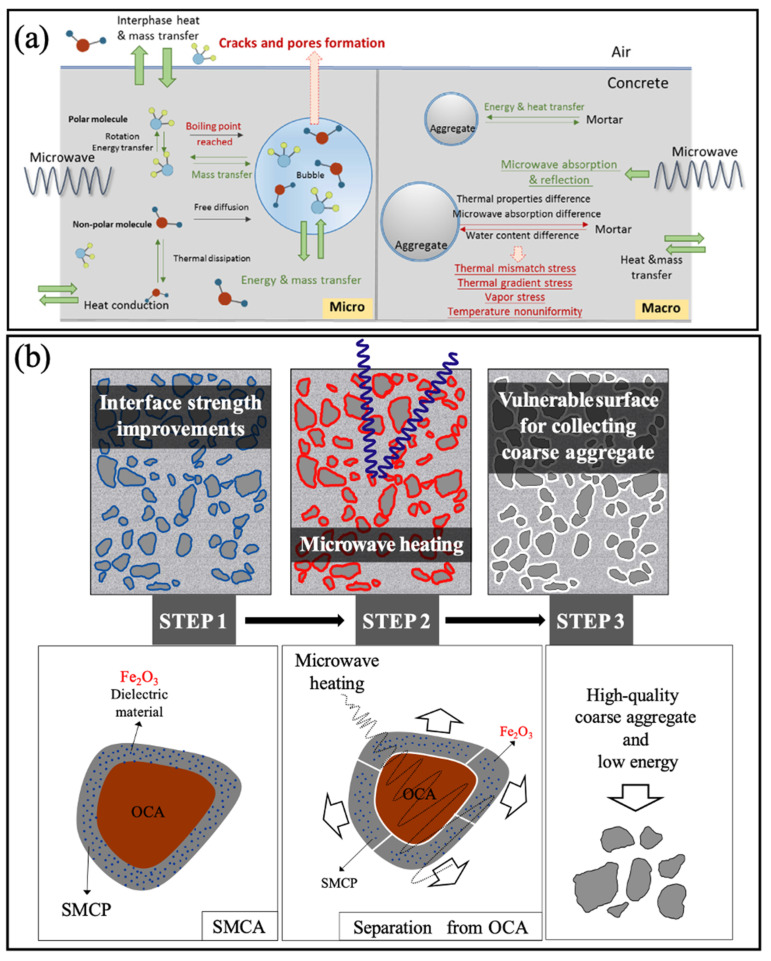
Modification of recycled aggregates via microwaves: (**a**) mechanism of microwave treatment (energy, heat, and mass transfer) [36]; (**b**) schematic diagram of microwave treatment method [37].

To mitigate the shortcomings associated with traditional thermal treatment, scholars have adopted microwave heating technology (see Figure 3). This innovative approach capitalizes on the differences in temperature trends, electrical conductivity, and physical properties among the various components to produce corresponding stress gradients. This, in turn, induces fractures in the targeted components while sparing the desired components (aggregate portion). This method addresses the drawbacks inherent in traditional thermal treatment [33,34]. Furthermore, during the treatment of recycled aggregates using microwave heating technology, thermal stress is generated specifically within the hardened cement paste, particularly in the interface transition zone connecting the aggregate and hardened cement paste. This leads to delamination between the aggregate and surface-hardened cement paste, thereby achieving the strengthening objectives [35]. In comparison to mechanical and acid treatment methods, this approach offers advantages such as shorter treatment times and higher efficiency, while consuming less energy compared to traditional thermal treatment, ultimately yielding more satisfactory results.

#### 3.1.4. Thermal Grinding Treatment

Thermal grinding treatment represents a hybrid approach that combines the efficacy of microwave thermal treatment with the mechanical grinding method. This methodology entails heating recycled aggregates to a high temperature prior to mechanical grinding. This preheating step causes the hardened paste on the aggregate surface to weaken due to the high-temperature decomposition of its internal components. Subsequently, mechanical grinding is applied to these high-temperature aggregates, effectively cleaning the surface impurities and yielding strengthened coarse and fine aggregates.

Thermal grinding treatment can be further categorized into thermal grinding and selective thermal grinding. In the case of thermal grinding, the high temperatures induce phase changes in the hardened paste, aggregates, transition zones, and microcracks of recycled aggregates due to temperature variations, leading to a degradation in performance. Consequently, when utilizing thermal grinding to remove adhered mortar from the aggregate surface, high temperatures create weak fracture processing zones on the aggregate, adversely affecting its performance. Therefore, selective thermal grinding emerges as a preferable option when aiming to produce high-quality recycled aggregates. Experimental studies conducted by Ma et al. [38] have demonstrated that, when mechanical treatment is adequate, hardened cement paste can be effectively removed at moderate temperatures ranging between 250 and 300 °C, yielding recycled aggregates with apparent densities that approximate those of natural aggregates. Similarly, Xiao et al. [39] achieved increased crushing capacity and aggregate release through microwave weakening treatment, followed by mechanical grinding, thereby validating the feasibility of thermal grinding treatment. In comparison to the thermal treatment and mechanical grinding methods, this hybrid approach amalgamates their strengths while compensating for their weaknesses, ultimately yielding superior strengthening effects.

#### 3.1.5. Autogenous Cleaning Process for Aggregate Treatment

**Figure 4:**
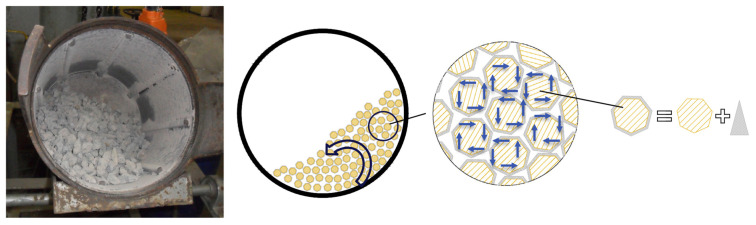
Autogenous cleaning of recycled aggregates [40].

The autogenous cleaning process entails the placement of recycled aggregates within a rotating milling drum, facilitating their interaction through collisions. This interaction achieves the detachment of hardened cement paste from the surfaces of the recycled aggregates. Subsequent to the strengthening phase, ultrasonic cleaning is employed to eliminate loose particles and impurities adhering to the aggregate surfaces as a result of these collisions, thereby completing the enhancement process (see Figure 4).

Pepe et al. [40] conducted comprehensive performance assessments on aggregates subjected to the advanced autogenous cleaning process following the crushing and recycling procedures. Their findings revealed a notable enhancement in aggregate performance. Furthermore, they observed that increasing the quantity of abrasive particles and augmenting the drum dimensions within the autogenous process could yield more pronounced effects in practical applications. However, it is crucial to acknowledge that mechanical treatment, during the collision phase, may also induce microcracks within the recycled aggregates themselves, potentially resulting in a decrement in the performance of recycled concrete. This underscores the need for a meticulous balance between purification efficiency and aggregate integrity to optimize concrete performance.

#### 3.1.6. Removing Adhered Mortar: Evaluation of the Performance

This section presents a detailed summary of the performance enhancement achieved through various techniques aimed at removing adhered mortar from aggregates, as well as the resultant impacts on the mechanical properties of cement-based materials. Table 1 provides an exhaustive list of key performance indicators, including water absorption, Los Angeles abrasion loss, relative density, apparent density, and both compressive and flexural strengths of recycled concrete.

When aggregates were subjected to acid immersion treatment, particularly HCl immersion, a notable reduction in water absorption ranging from 23.2% to 40.4% was observed. Additionally, there were decreases in both the Los Angeles abrasion loss and relative density. Importantly, concrete formulated using these treated aggregates exhibited strengths comparable to those formulated with natural aggregates, thus validating the efficacy of acid immersion treatment in strengthening aggregates. Regarding thermal and mechanical treatment methods, enhancements in the pertinent performance indicators of recycled aggregates were observed as the intensity of the strengthening conditions increased. Specifically, water absorption decreased by 50% to 80.7%, and the apparent density approached that of natural aggregates. These data-driven findings affirm the feasibility of these methods. However, it is important to note that the majority of research in this area has centered on improving the physical properties of aggregates, with relatively limited exploration into the effects of strengthened aggregates on concrete strength. On the other hand, the autogenous cleaning method for aggregate treatment resulted in performance improvements as the duration of treatment increased. While not as potent as thermal and mechanical treatments, it nevertheless demonstrated commendable enhancements in water absorption, bulk density, and the strength of concrete formulated after strengthening. In conclusion, among the diverse techniques for removing adhered mortar, thermal grinding treatment for strengthening stands out due to its superior processing efficiency. This method is capable of optimizing the pertinent performance indicators of recycled aggregates to their utmost potential, thereby fulfilling the objectives of enhancement.

### 3.2. Strengthening Adhered Mortar

Compared to removing adhered mortar, strengthening it exhibits superior improvement effects on the mechanical and physical properties of recycled aggregates as well as the comprehensive performance of freshly mixed concrete produced with these aggregates. Effective methods for strengthening adhered mortar include (1) microbial mineralization enhancement; (2) carbonation treatment enhancement; (3) modification enhancement with mineral admixtures; and (4) polymer impregnation enhancement.

#### 3.2.1. Microbial Mineralization Enhancement

In 1973, Boquet et al. [41] first discovered the induction of mineralization by soil bacteria, resulting in carbonate deposition. They proposed that, under suitable external conditions, many bacteria can also achieve microbial mineralization and deposition reactions. Subsequently, microbial-induced carbonate precipitation (MICP) technology began to develop gradually. MICP technology primarily refers to the utilization of microbial biological control and induction processes to promote the precipitation of calcium carbonate in different environments.

In 1989, Lowenstam et al. [42] found that microbial mineralization deposition can be broadly divided into microbial-autonomous deposition and microbial-induced deposition. Microbial-autonomous deposition mainly involves the nucleation and formation of mineral particles, leading to deposition. In contrast, microbial-induced deposition occurs when specific bacteria, under certain environmental conditions, induce mineralization and produce deposition. Over decades of exploration and development, mineralization deposition technology has gradually improved and is now widely applied in soil solidification, the improvement of geotechnical properties, strengthening materials, and remediation materials. Compared to traditional methods, mineralization deposition methods have mild reaction conditions, good compatibility, and weak negative effects and do not disrupt ecological balance or harm the environment.

The basic principle of MICP technology is to utilize the bonding properties of calcium carbonate and the stability of calcite produced by microbial mineralization to solidify and fill soil or materials. The reaction types are divided into three categories: urease type, metabolic type, and carbonic anhydrase type [43]. Each type of microbial mineralization conversion has its own advantages and disadvantages, with reaction equations as shown in Equations (1)–(7).

Urease-type microbial reaction equations:

(1) $$\begin{array}{c} \mathrm{CO}{{(NH}_{2})}_{2}+2H_{2}O\to 2{NH}_{4}^{+}+{CO}_{3}^{2-} \end{array}$$

(2) $$\begin{array}{c} \mathrm{cell}+{Ca}^{2+}\to \mathrm{cell}-{Ca}^{2+} \end{array}$$

(3) $$\begin{array}{c} \mathrm{cell}-{Ca}^{2+}+{CO}_{3}^{2-}\to \mathrm{cell}-{CaCO}_{3}↓ \end{array}$$

Metabolic microbial reaction equations:

(4) $$\begin{array}{c} {CaC}_{6}H_{10}O_{6}+6O_{2}\to {CaCO}_{3}↓+5CO_{2}↑+5H_{2}O \end{array}$$

(5) $$\begin{array}{c} 5\mathrm{Ca}{\left(OH\right)}_{2}+5CO_{2}\to 5{CaCO}_{3}↓+5H_{2}O \end{array}$$

Carbonic anhydrase-type microbial reaction equations:

(6) $$\begin{array}{c} H_{2}O+CO_{2}\to HCO_{3}^{-}+H^{+} \end{array}$$

(7) $$\begin{array}{c} HCO_{3}^{-}+{Ca}^{2+}+{OH}^{-}\to {CaCO}_{3}↓+H_{2}O \end{array}$$

Among the three reaction types, the urease-type microbial mineralization reaction is characterized by easy control and high efficiency in the mineralization process, but it also generates ammonia, causing air pollution. In metabolic mineralization reactions, the process is difficult to control and has lower efficiency, but no harmful by-products are generated during the reaction. Carbonic anhydrase-type reactions are easy to control and efficient and do not produce environmentally harmful by-products, making them more ecologically friendly.

**Figure 5:**
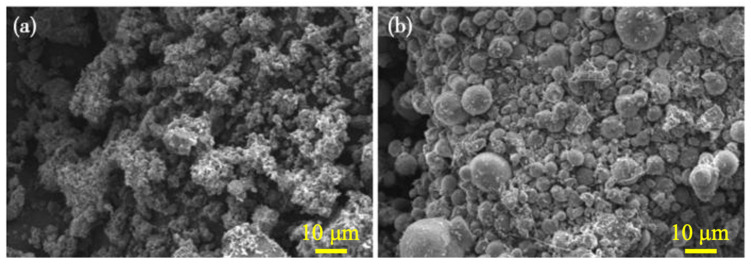
Micrograph of recycled concrete aggregates (RCA): (**a**) RCA without MICP treatment; (**b**) RCA with MICP treatment [44].

Currently, researchers are engaged in an extensive investigation of strengthening techniques involving the application of microbial mineralization within recycled aggregates. Qiu et al. [44] implemented the Microbially Induced Calcite Precipitation (MICP) technology to perform surface modification on recycled aggregates, focusing on determining the optimal cultivation milieu for microorganisms and precipitation parameters. Figure 5 illustrates the microstructural characteristics and superficial morphology of untreated RCA and RCA treated with MICP technology. As depicted in Figure 5a, the untreated RCA surface exhibits a porous topology with considerable irregularity. Conversely, the surface of MICP-treated RCA, as shown in Figure 5b, reveals the formation of spherical calcite crystals, resulting in a more consolidated and ordered structure compared to Figure 5a. An analysis via Energy-Dispersive X-ray Spectroscopy (EDX) suggests that the superficial crystals consist of calcium carbonate precipitated by bacteria. These findings affirm the feasibility of MICP technology in reinforcing recycled aggregates, leading to substantial improvements in their material properties.

Khushnood et al. [45] conducted a study considering the survival niche of microorganisms, local environmental conditions, and economic factors. They employed a method involving the immobilization of bacteria on recycled coarse aggregates and virgin fine aggregates for direct induction, aiming to ensure the viability of bacteria introduced into concrete. This approach serves multiple purposes, including cost reduction, conservation of natural resources, and advancement of sustainable development goals. Furthermore, they validated the feasibility and environmental benignity of microbial-modified recycled coarse aggregates. Feng et al. [46] leveraged streptococcus pasteurianus to catalyze the production of calcium carbonate for the strengthening of recycled fine aggregates, comprehensively demonstrating the positive effects of mineralization deposition technology. A previous study [47] compared the strengthening efficacy of ureolytic and non-ureolytic bacteria on recycled aggregates. The results indicated that both types of bacteria could ameliorate the material properties of recycled aggregates, with ureolytic Bacillus species exhibiting superior mineralization potential. This finding reaffirms the rapid kinetics and high efficiency associated with ureolytic microbial reactions. Salman Rais et al. [48,49,50] investigated the impact of microbial mineralization technology on the performance of recycled aggregate concrete. They discovered that the calcium carbonate precipitation generated by MICP technology could effectively fill internal pores and voids within the concrete, resulting in a denser structure and significant enhancement of mechanical strength, durability, and other pertinent properties. In conclusion, microbial mineralization deposition technology emerges as a promising and environmentally benign approach. However, it remains susceptible to various conditions and is still in the experimental validation phase, necessitating further research and refinement to fully harness its potential.

#### 3.2.2. Accelerated Carbonation Treatment for the Strengthening of Recycled Aggregates

Carbonation treatment represents a sophisticated method to enhance the properties of cement-based materials by utilizing CO_2_ to refine the microporous structure within the cement paste matrix [51,52,53,54,55,56]. This refinement process leads to improvements in surface hardness, compressive strength, and durability. Analogously, when applied to recycled aggregates, carbonation treatment can effectively refine the pore structure of the cement paste adhering to the aggregate surface, thereby enhancing the mechanical and physical attributes of these aggregates. In the realm of aggregate strengthening, carbonation can be elucidated as a chemical reaction involving the dissolution of CO_2_ in water, which subsequently reacts with Ca(OH)_2_ present in the cement mortar adhering to the aggregate surface. This reaction results in the precipitation of CaCO_3_, which fills and strengthens the pores on the mortar surface. Consequently, the porosity of the adhering mortar is reduced, leading to enhanced aggregate performance. Furthermore, under moist conditions, hydrated calcium silicate and calcium aluminate products within the adhering mortar also undergo carbonation reactions with CO_2_, yielding CaCO_3_ precipitation, aluminates, and silicates [57,58], as described by Equations (8)–(11).

(8) $$\begin{array}{c} {Ca\left(OH\right)}_{2}+{CO}_{2}\to {CaCO}_{3}↓+H_{2}O \end{array}$$

(9) $$\begin{array}{c} 2{SiO}_{2}\cdot 3\mathrm{CaO}\cdot 3H_{2}O+3{CO}_{2}\to 2{SiO}_{2}+{3CaCO}_{3}↓+{3H}_{2}O \end{array}$$

(10) $$\begin{array}{c} 4\mathrm{CaO}\cdot {Al}_{2}O_{3}\cdot 13H_{2}O+4{CO}_{2}\to 2\mathrm{Al}({OH)}_{3}+{4CaCO}_{3}↓+{10H}_{2}O \end{array}$$

(11) $$\begin{array}{c} \mathrm{Ca}{CO}_{3}+H_{2}O+2{CO}_{2}\to \mathrm{Ca}{\left(HCO_{3}\right)}_{2} \end{array}$$

As the carbonation reaction progresses, the consumption of Ca(OH)_2_ results in a gradual decrease in the pH value of the cement mortar to below 9.0. This pH reduction can potentially compromise the protective oxide film on the outer surface of internal reinforcing steel bars in concrete, thereby increasing the susceptibility of steel corrosion and compromising concrete durability. However, from a strengthening perspective, carbonated recycled aggregates exhibit improved water absorption characteristics and apparent density. Additionally, they effectively augment the microhardness of the old interfacial transition zone, significantly enhancing the physical properties of the recycled aggregates [59,60].

Zhan et al. [57,58,61] conducted research by formulating concrete using CO_2_-carbonated modified recycled aggregates. Their findings revealed that CO_2_-carbonated hardened cement paste can be utilized to produce structural concrete with durability comparable to that of concrete made with natural aggregates. Upon validating the efficacy of carbonation treatment in reinforcing recycled aggregates, they further investigated the optimal conditions for achieving the desired carbonation effects. Through a rigorous process of single-variable adjustment and control, the optimal carbonation conditions were determined. Prior to carbonation treatment, the recycled aggregates were maintained in an environment with a controlled temperature of (25 ± 3) °C and a relative humidity of (50 ± 5)%. The curing chamber was evacuated using an air pressure of 0.06 MPa, and the aggregates were placed inside. Subsequently, CO_2_ was injected into the chamber at a constant pressure of up to 0.5 MPa. The aggregates were then cured for seven days in an environment with a CO_2_ concentration of 100%. This process led to a significant enhancement in the properties of the recycled aggregates [62,63,64].

It is important to note that the content of Ca(OH)_2_ plays a pivotal role in the carbonation strengthening process. For recycled aggregates with low Ca(OH)_2_ content, the certainty of pore filling via carbonation treatment is uncertain. Although soaking the aggregates in a Ca(OH)_2_ solution prior to carbonation treatment can be considered, this approach may still result in asynchronous and incomplete carbonation of the aggregates. Therefore, careful consideration and precise control of the carbonation conditions are essential to ensure optimal strengthening effects.

#### 3.2.3. Modification of Recycled Aggregates via Mineral Admixtures and Nanomaterials

In contrast to natural aggregates, recycled aggregates exhibit a porous structure and a high degree of heterogeneity. This distinct microstructural characteristic has prompted researchers to propose a method involving the infiltration of mineral admixtures into the pore spaces of recycled aggregates. The objective is to leverage the secondary hydration products generated by these admixtures to fill and improve the microstructure of microcracks, pores, and old interfacial transition zones (ITZs) within recycled aggregates, thereby enhancing their overall performance.

To validate the efficacy of mineral admixtures in improving the defects of recycled aggregates through secondary hydration reactions, researchers conducted experiments involving the application of various types of mineral admixtures to the surface of recycled aggregates. The results indicated that the incorporation of these admixtures effectively filled pores and microcracks on the surface of the aggregates. Furthermore, the ITZ between the aggregates and the hardened cement paste was improved. Notably, the use of composite mineral admixtures resulted in a more densified ITZ, significantly enhancing the strength and durability of the recycled aggregates [65,66]. Despite its potential benefits, the use of silica fume poses certain challenges. Silica fume tends to agglomerate, forming clusters ranging from 10 to 1000 mm in size. The addition of agglomerated silica fume to recycled concrete results in minimal changes in strength. By mathematical models provided by a previous study, the optimization of concrete compositions for partial replacement of slag cement with brick powder is up to 30%, and natural sand with recycled sand is up to 100% with the addition of an alkaline activator in the range of 0.5–1% of the cement content. The mixture results in a sustainable, alkali-activated high-strength self-compacting recycling concrete that is environmentally friendly [67]. This is attributed to the inability of conventional admixtures to completely decompose the agglomerated silica fume. As a result, the cores of these agglomerates remain un-hydrated, becoming the weakest links in the paste mixture. This leads to a decrement in the physical filling effect and reactivity index of the agglomerated silica fume, thereby failing to improve the microstructure and mechanical properties of the hardened cement paste.

**Figure 6:**
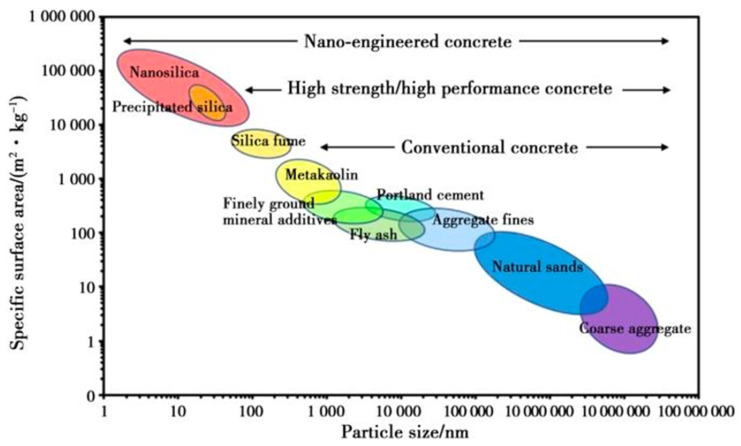
Particle size and specific surface area of typical components constituting concrete.

**Figure 7:**
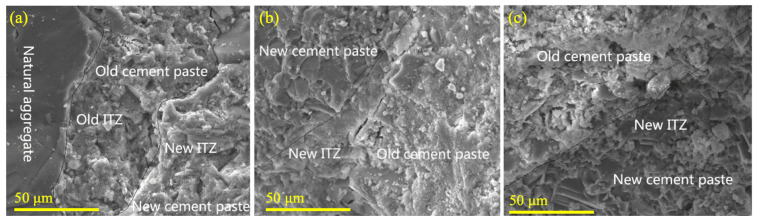
Micrographs of recycled aggregate concrete (RAC): (**a**) plain RAC; (**b**) RAC treated with nano-limestone; (**c**) RAC treated with nano-silica.

Advancements in research have highlighted the superior advantages of using nanomaterials for the modification of recycled aggregates. Nanoparticles, due to their high flowability and continuous pore-filling capabilities, can achieve a high degree of compactness (as illustrated in Figure 6). This results in a significant enhancement in the performance of recycled aggregates. The incorporation of nano-mineral admixtures, such as nano-SiO_2_ [68,69] particles coated onto the surface of aggregates, facilitates secondary reactions with hydration products in the attached mortar. These reactions generate products that fill the micropores of the aggregates. The encapsulation of the paste by nanoparticles and the filling of micro-porosity in the ITZ by secondary hydration products effectively improve the nanomechanical properties of the ITZ between aggregates and old mortar. Consequently, the physical properties of the recycled aggregates are enhanced. Similarly, the application of nano-CaCO_3_ involves the formation of secondary hydration products, such as C-S-H gel, and the use of inert nanoparticles to fill microcracks in aggregates, resulting in a denser microstructure of the cement-based paste [70,71,72,73]. Figure 7 demonstrates the microstructural improvements in the old and new ITZs achieved through the use of nano-SiO_2_ and nano-limestone [74]. In conclusion, the enhancement of recycled aggregates through the use of mineral admixtures and nanomaterials represents a significant advancement in the field of materials science and engineering. By leveraging the unique properties of these materials, researchers have been able to address the challenges associated with recycled aggregates, thereby improving their performance and expanding their potential applications.

#### 3.2.4. Polymer Impregnation Techniques for the Strengthening of Recycled Aggregates

Given the inherent deficiencies in the properties of recycled aggregates, researchers have embarked on an innovative approach to enhance their performance. This involves utilizing the filling capabilities of polymers and their unique characteristics in cement hydration reactions to improve key parameters such as water absorption rate, crushing resistance, and microstructure of recycled aggregates. This, in essence, bolsters the overall properties of recycled aggregate concrete.

The majority of research endeavors in the realm of polymer modification have centered around the utilization of polyvinyl alcohol (PVA) as a strengthening agent for recycled aggregates [75,76,77]. The findings from these studies uniformly reveal a substantial increase in the apparent density of the modified recycled aggregates, accompanied by a decrease in both water absorption rate and permeability. Notably, PVA has demonstrated its prowess not only in filling pores and cracks within the aggregates but also in significantly enhancing the interfacial bonding strength between the recycled aggregates and the surrounding hardened cement paste. This enhancement translates into improved performance metrics for both recycled aggregate mortar and recycled aggregate concrete.

Furthermore, the application of polymer treatment extends beyond mere physical improvement, offering enhancements in water absorption rate, workability, and durability of recycled aggregates. However, it is crucial to acknowledge that the polymer impregnation strengthening process primarily involves transforming the failure mechanisms of the aggregates rather than achieving a fundamental transformation in their material properties. Consequently, while the treated recycled aggregates do exhibit some degree of improvement in their physical properties and mechanical strength, these enhancements are relatively modest when compared to their untreated counterparts.

In summary, the advanced polymer impregnation techniques represent a significant stride towards optimizing the performance of recycled aggregates. However, continued research is warranted to explore potential enhancements in the treatment process that could lead to more substantial improvements in the properties of recycled aggregates, ultimately contributing to the broader adoption and sustainability of recycled aggregate concrete in the construction industry.

#### 3.2.5. Adhesive Mortar Strengthening: Performance Evaluation

Table 2 documents the enhancement effects on aggregate performance parameters and their resultant impacts on the mechanical attributes of cementitious materials subsequent to the application of diverse mortar strengthening methodologies. These parameters encompass water absorption coefficient, Los Angeles abrasion resistance, crushing strength index, relative density, and apparent density, among others. The incorporation of microbial mineralization and nano-mineral admixture modification techniques in reinforcing recycled aggregates has led to marked improvements across these performance indicators, with microbially treated aggregates surpassing natural aggregates in terms of specific properties.

In the context of carbonation strengthening, treated aggregates exhibited a substantial reduction in water absorption rates ranging from 18% to 48%, coupled with improvements in both crushing strength index and apparent density. Nevertheless, when benchmarked against natural aggregates, these enhanced properties still exhibited a slight disparity. Polymer impregnation, as a strengthening technique for recycled aggregates, resulted in a pronounced enhancement in water absorption rates, approaching those of natural aggregates. However, its efficacy in augmenting mechanical properties was relatively constrained, thereby limiting its broader applicability. Currently, the preponderance of research endeavors has been directed towards reinforcing recycled aggregates using monolithic methodologies, with the resultant strengthening effect often falling shy of anticipated benchmarks. In light of this, future investigative endeavors could profitably explore the potential of composite strengthening techniques, employing a synergy of diverse methodologies to optimize strengthening outcomes.

In conclusion, a comparison of several strengthening techniques for adhesive mortar reveals that microbial mineralization treatment of recycled aggregates exhibits the highest processing efficacy, ultimately achieving superior strengthening efficacy (see Table 3). This advanced terminology and refined logical flow serve to enhance the academic rigor and depth of the analysis.

## 4. Nano-Titanium Dioxide-Modified Recycled Aggregates: Mechanisms, Performance, and Sustainability Implications

The integration of nanotechnology into construction material science has revolutionized the valorization of RCAs, particularly through the application of nano-titanium dioxide (nano-TiO_2_) [88,89,90]. Conventional remediation strategies, such as carbonation curing or polymer impregnation, offer marginal improvements but fail to address nanoscale defects or confer multifunctionality. In contrast, nano-TiO_2_ modification has emerged as a paradigm-shifting approach, leveraging the nanomaterial’s unique physicochemical properties to simultaneously enhance RCA performance and imbue novel functionalities. The efficacy of nano-TiO_2_ as a modifier stems from its dual role as a pore-filling agent and a photocatalytic activator. Anatase-phase TiO_2_ nanoparticles (<50 nm) exhibit high surface reactivity, enabling their penetration into RCA micropores (1–10 μm diameter). This reaction densifies ITZs, reducing porosity and enhancing aggregate-cement bonding strength, as validated by nanoindentation and scanning electron microscopy (SEM) analyses [91,92]. Concurrently, nano-TiO_2_’s photocatalytic activity under ultraviolet (UV-A/B) irradiation generates hydroxyl radicals (•OH) and superoxide anions (O_2−_), which degrade organic pollutants (e.g., NO_x_ and VOCs) via advanced oxidation processes (AOPs). Field studies of photocatalytic pavements incorporating TiO_2_-modified RCAs report NO_x_ abatement efficiencies under ambient solar exposure, demonstrating their potential for urban air quality remediation.

Three principal strategies dominate the nano-TiO_2_ functionalization of RCAs, each with distinct mechanisms and outcomes. (a) Surface Coating via Sol-Gel Deposition: RCAs are immersed in TiO_2_ sols (1–5 wt% concentration) [92] and subjected to thermal calcination (300–500 °C), forming a mesoporous TiO_2_ layer (5–20 μm thickness). This technique reduces water absorption by 18–22% and enhances abrasion resistance by 30%, though energy-intensive calcination raises sustainability concerns. (b) In situ Dispersion in Cementitious Matrices: Direct incorporation of nano-TiO_2_ (0.5–3% by cement weight) during concrete mixing accelerates early-age hydration kinetics, as evidenced by isothermal calorimetry. Optimal dosages (1.5–2%) yield 28-day compressive strengths of 35–40 MPa—comparable to that of natural aggregates—while reducing chloride diffusion coefficients to <1.5 × 10^−12^ m^2^/s, a 50–60% improvement over unmodified RCAs. (c) Covalent Grafting via Silane Coupling Agents: Functionalization with 3-aminopropyltriethoxysilane (APTES) enables covalent bonding between TiO_2_ nanoparticles and RCA surfaces. This method enhances photocatalytic efficiency compared to physical coatings, as silane linkages prevent nanoparticle leaching under hydraulic pressure.

The synergistic effects of nano-TiO_2_ modification manifest across mechanical, durability, and functional domains. In synthesizing these advancements, nano-TiO_2_-modified RCAs represent not merely incremental improvement but also a redefinition of waste-derived construction materials—transforming them into high-performance, multifunctional assets aligned with the United Nations Sustainable Development Goals (SDGs) 9 (Industry, Innovation, and Infrastructure) and 11 (Sustainable Cities). The technology’s success hinges on transdisciplinary collaboration, merging material innovations with environmental economics and policy design to overcome residual technical and market barriers.

## 5. Existing Problems and Suggestions

There are numerous methods for reinforcing recycled aggregates, yet several pressing issues remain to be addressed. These issues are primarily manifested in the following aspects related to the removal of adhered mortar during the preparation of recycled aggregates:

(1) Acid treatment strengthening: After treating recycled aggregates with acid solutions, a significant amount of water is required for rinsing to eliminate chemical impurities. Furthermore, the chemically contaminated water needs to be appropriately recycled to protect the environment from pollution. This process is cumbersome and may potentially cause secondary environmental damage. (2) Mechanical grinding treatment strengthening: During the crushing of adhered cement paste, numerous microcracks and micropores are generated on the aggregate surface, affecting the inherent properties of the recycled aggregates. Additionally, this method has defects such as high energy consumption and incomplete removal. (3) Thermal treatment strengthening: Thermal treatment strengthening is divided into traditional thermal treatment and microwave heating treatment strengthening. Although the latter improves upon traditional methods by addressing the inability to selectively fracture components and release multiphase materials, the unit energy consumption required for thermal treatment remains high, accompanied by substantial CO_2_ emissions, necessitating further improvements. (4) Thermal grinding treatment strengthening: This method has similar defects to thermal treatment strengthening, including high CO_2_ emissions and energy consumption. (5) Physical autogenous cleaning process strengthening: During the crushing process, mutual collisions between aggregates are utilized to break the hardened cement paste on the surface. However, this process can damage the aggregates themselves, leading to reduced physical properties.

In terms of reinforcing adhered mortar during the preparation of recycled aggregates, we have the following (see Table 4). (1) Microbial mineralization strengthening: This method has numerous influencing factors and exhibits poor stability in practical applications, posing significant challenges and requiring further research. (2) Carbonation treatment strengthening: The carbonation effect varies depending on the type of recycled aggregate. For aggregates with low Ca(OH)_2_ content, incomplete carbonation often occurs, leading to unstable strength. Additionally, the consumption of Ca(OH)_2_ during the carbonation process weakens the oxide film of internal steel bars in newly mixed concrete, further contributing to unstable strength. (3) Mineral admixture modification strengthening: Most studies use nano-silica or silicon powder as mineral admixtures. During the strengthening process, silicon powder tends to form agglomerates ranging from 10 to 1000 μm, making it difficult to disperse and reducing its physical filling effect and reactivity index. This hinders the complete decomposition needed to improve the microstructure and mechanical properties of the hardened cement paste on the surface of recycled aggregates. Furthermore, various factors such as the dosage of nano-mineral admixtures affect the stability of strengthening. Therefore, further efforts are needed to achieve breakthroughs in the application of nanotechnology to reinforce recycled aggregates. (4) Polymer impregnation strengthening: Although this method shifts primary interface failure to mixed interface failure, the physical properties of the aggregates are not significantly improved, limiting the strengthening effect.

## 6. Conclusions

The current array of strengthening methodologies exhibits notable efficacy in the recycling and reuse of recycled aggregates. Despite the inherent strengths and weaknesses associated with each approach during the strengthening process, it is undeniable that these technologies provide substantial technical support for the utilization of recycled aggregates. Relatively speaking, they have demonstrated commendable outcomes and possess promising application prospects in terms of strengthening the adhered mortar on the aggregate surface. Among these strengthening techniques, the microbial mineralization carbonate precipitation method stands out for its relatively superior effectiveness in enhancing aggregate performance, while simultaneously avoiding secondary pollution during the process. Although the mineralization deposition technology is still in its nascent stages and has not yet been widely adopted, advancements in society and technological progress suggest that this method holds promise as an environmentally friendly and pollution-free strengthening strategy.

Another technique that exhibits significant efficacy in improving the performance of recycled aggregates is the nano-mineral admixture modification approach. While the aggregates treated with this method may not match the quality of natural aggregates, their performance improvements surpass those achieved through several other strengthening methodologies. However, it is crucial to exercise stringent control over the experimental conditions to ensure optimal results. Currently, research efforts are predominantly concentrated on refining individual strengthening methods, with limited exploration into composite strengthening strategies involving two or more methodologies. Consequently, identifying appropriate strengthening techniques to strengthen aggregates, thereby enhancing their performance to match or even surpass that of natural aggregates, remains a pressing concern for concrete researchers. Addressing this challenge is crucial for mitigating the shortage of natural aggregates and reducing the arbitrary landfilling of construction waste.

## Figures and Tables

**Table 1:** Comparison of various treatment methods to remove adhered mortar.

Method	Process Description	Key Advantages	Challenges/Limitations
Acid Treatment	Immersion in acidic solutions (e.g., CH_3_COOH, HCl, H_2_SO_4_, and H_3_PO_4_) to dissolve hardened cement paste.	Dissolves rigid surface mortar, improving aggregate quality. Reduces chloride ion (Cl^−^) content with thermal pre-treatment.	Generates chemically contaminated wastewater (sulfates/chlorides). High water consumption for washing. Environmental risks if wastewater is untreated.
Mechanical Grinding	High-speed rotating gears or ball mills induce shear stress to detach adhering mortar.	Efficient removal of surface paste. Ball mills show superior efficacy over conventional grinding.	Causes microcracks and increased porosity on aggregates. High energy consumption and heat generation.
Thermal Treatment	Heating aggregates (~500 °C for 2 h) to fracture surface paste via thermal stress.	Microwave heating reduces energy use and treatment time. Targets hardened paste while preserving aggregates.	Traditional heating induces microcracks in aggregates. High energy demands for conventional thermal methods.
Thermal Grinding	Combines microwave preheating (250–300 °C) with mechanical grinding to weaken mortar.	Synergizes thermal and mechanical effects for enhanced mortar removal. Yields densities close to natural aggregates.	High temperatures may degrade aggregate performance if uncontrolled. Complex temperature management required.
Autogenous Cleaning	Aggregates collide in a rotating drum to dislodge mortar, followed by ultrasonic cleaning.	Improves aggregate performance without external chemicals. Scalable with adjustable drum size and abrasive particles.	Collision-induced microcracks may reduce concrete durability. Requires balancing purification and structural integrity.

**Table 2:** Performance evaluation of various methods of removing old mortar.

Modification	Parameters	Type	A	B	C	D	E	F
Acid Treatment (HCl) [28,78,79,80,81]	-	NA	1.83	28	2.67	-	23.27	2.95
	With	RA	5.2	31	2.58	-	22.41	2.73
	Withous	RA	3.1	29.5	2.47	-	23.06	2.94
Mechanical Grinding Treatment/Heat Treatment [38,82,83,84]	-	NA	-	-	-	2.55	-	-
	20 °C	8/16 mm RA	4.3	-	-	2.65	-	-
	300 °C	8/16 mm RA	3.4	-	-	2.65	-	-
	600 °C	8/16 mm RA	1.9	-	-	2.74	-	-
	Before Modification	8/16 mm RA	5.7	-	-	2.32	-	-
	5 min	8/16 mm RA	3	-	-	2.52	-	-
	20 min	8/16 mm RA	1.9	-	-	2.55	-	-
	40 min	8/16 mm RA	1.1	-	-	2.6	-	-
Autogenous Cleaning Treatment [40,78,79,85]	-	NA	3.39	-	-	2.46	33.02	3.85
	Before Modification	4.75/9 mm RA	11.94	-	-	1.95	27.5	3.36
	10min	4.75/9 mm RA	6.06	-	-	2.22	-	-
	15min	4.75/9 mm RA	5.56	-	-	2.26	29.92	3.71

Note: A—Water Absorption Rate (%); B—Los Angeles Abrasion Loss (%); C—Relative Density g·cm^3^; D—Bulk Density g·cm^3^; E—28-day Compressive Strength (MPa); F—28-day Flexural Strength (MPa).

**Table 3:** Performance evaluation of various methods of strengthening old mortar.

Modification		Type	A	B	C	D	E
Microbial mineralization and nano silica treatment [43,44,83,86]	-	NA	0.8	-	20.06	2.62	-
	Before	RA	1.4	-	26.51	2.7	-
	After	RA (nano-SiO_2_)	0.9	-	-	2.7	-
	After	RA (ureolytic bacteria)	0.5	-	-	2.87	-
	After	RA (non-ureolytic bacteria)	0.5	-	-	2.89	-
Carbonation treatment [54,57,60,87]	-	NA	2.35	-	-	-	2.7
	Before	RA	8.06	-	18.6	-	2.53
	After	RA	5.78	-	16.9	-	2.65
Polymer modification [75,76,77]	-	NA	0.7 ± 0.1	23~24	-	-	-
	Before	12~20 mm RA	4.5 ± 0.2	27 ± 2	-	-	-
	After	12~20 mm RA	0.7	21 ± 1	-	-	-

Note: NA—Natural Aggregate; RA—Recycled Aggregate; A—Water Absorption Rate (%); B—Los Angeles Abrasion Loss (%); C—Crushing Index (%); D—Relative Density g·cm^3^; E—Bulk Density g·cm^3^.

**Table 4:** Comprehensive comparison among various modification methods.

Strengthening Method		Description	Challenges/Considerations
Removal of Adhered Mortar	Acid Treatment	Extensive rinsing required to eliminate chemical impurities	Environmental impact of contaminated water, cumbersome process
	Mechanical Grinding Treatment	Crushing adhered cement paste generates microcracks and micropores	High energy consumption, incomplete removal of adhered mortar
	Thermal Treatment	Encompasses traditional and microwave heating methods	High unit energy consumption, significant CO_2_ emissions
	Thermal Grinding Treatment	Inherits energy consumption and emission issues from thermal treatment	Complex equipment
	Physical Self-Cleaning Process	Aggregate-to-aggregate collisions fragment surface-hardened cement paste	Potential damage to aggregate, detracting from physical performance
Strengthening of Adhered Mortar	Microbial Mineralization	Influenced by numerous variables, leading to inconsistent performance	Further research required to address challenges
	Carbonation Treatment	Efficacy varies depending on type of recycled aggregate	Low Ca(OH)_2_ content results in incomplete carbonation, weakens oxide film of internal steel strengthening
	Mineral Admixture Modification	Nano-silica and silicon powder tend to aggregate into particles, impeding dispersion	Incomplete decomposition hinders improvement of microstructure and mechanical properties, dosage significantly influences stability
	Polymer Impregnation	Shifts primary interface failure to mixed interface failure	Limited enhancement of physical properties of aggregates

## Data Availability

No new data were created or analyzed in this study. Data sharing is not applicable to this article.
